# ANXA1‐Derived Peptide Increases Melanoma Cell Sensitivity to Vemurafenib by Downregulating EphA2

**DOI:** 10.1111/jcmm.71310

**Published:** 2026-08-19

**Authors:** Juan Feng, Xiao‐Pu Huang, Ming Zhang, Wei Huang, Shan‐Shan Lu, Zhi‐Qiang Xiao, Zhi‐Hai Xie, Hong Yi

**Affiliations:** ^1^ Department of Pathology, the Second Xiangya Hospital Central South University Changsha Hunan China; ^2^ Department of Oncology, Xiangya Hospital Central South University Changsha Hunan China; ^3^ Research Center of Carcinogenesis and Targeted Therapy Xiangya Hospital, Central South University Changsha Hunan China; ^4^ The Higher Educational Key Laboratory for Cancer Proteomics and Translational Medicine of Hunan Province Xiangya Hospital, Central South University Changsha Hunan China; ^5^ Department of Oncology Xiangtan Central Hospital Xiangtan Hunan China; ^6^ Department of Otolaryngology Head and Neck Surgery Xiangya Hospital, Central South University Changsha Hunan China; ^7^ National Clinical Research Center for Geriatric Diseases (Xiangya Hospital of Central South University) Changsha Hunan China

**Keywords:** A11, EphA2, melanoma, sensitivity, vemurafenib

## Abstract

Vemurafenib (VEM) is a BRAF inhibitor that improves the prognosis of melanoma, but acquired resistance represents a key limitation to its clinical efficacy. This study investigated the potential of A11, an Annexin A1 (ANXA1)‐derived peptide, to enhance VEM efficacy in both VEM‐sensitive and VEM‐resistant melanoma cells. We evaluated the effects of A11 in melanoma through in vitro assays (including MTT, clonogenic survival, apoptosis, and cell cycle analysis) and in vivo xenograft models in nude mice. Furthermore, we mechanistically interrogated A11‐mediated suppression of EphA2 expression and the downstream pS897‐EphA2/AKT/ERK signalling pathway in resistant and parental cell lines via Western blotting and immunohistochemistry (IHC). Critically, our study demonstrates that A11 inhibits melanoma cell proliferation and reverses VEM resistance through EphA2 downregulation, consequently ablating this oncogenic pathway. This research highlights the promising application value of A11 in improving the efficacy of VEM treatment in melanoma, particularly in VEM‐resistant melanoma.

**Abbreviations**
A11: a peptide contains 11 amino acids of Annexin A1 at N‐terminal region from 20 to 30
ANXA1: Annexin A1
CPP: cell‐penetrating membrane peptide, YGRKRRQRRR
FBS: fetal bovine serum
IHC: immunohistochemistry
TCGA: The Cancer Genome Atlas
VEM: vemurafenib

## 1 Introduction

Cutaneous melanoma is one of the most aggressive cutaneous malignancies, and patients with metastatic melanoma have an estimated 5‐year survival rate of less than 10% [1]. A mutation in the BRAF gene is a common event that plays a key role in the development and progression of melanoma, providing the scientific foundation for the development of antitumour drugs that target BRAF mutations [2]. The BRAF (V600E) mutation is the most common mutation type, accounting for approximately 80% of all BRAF mutations, and it promotes the malignant transformation and progression of melanoma by activating MAPK signalling [2, 3]. Therefore, BRAF (V600E)‐specific inhibitors, such as VEM, have been developed and applied in clinical treatment, and they have achieved promising therapeutic outcomes [4, 5, 6, 7, 8]. Unfortunately, similar to other targeted agents, acquired therapy resistance remains the greatest obstacle for the durable efficacy of VEM in clinical practice [9, 10]. Consequently, elucidating the mechanisms of VEM resistance and developing novel strategies to enhance VEM therapeutic efficacy in melanoma patients represent critically important objectives.

The oncogenic roles of BRAF (V600E) depend on the activation of extracellular signal‐regulated kinase (ERK) signalling [3]. BRAF (V600E) is most easily activated by RAS, and subsequently activates MEK/ERK, which leads to the direct phosphorylation of downstream regulators and promotion of tumour proliferation [11]. As a specific inhibitor of BRAF (V600E), VEM preferentially suppresses the phosphorylation of MEK and ERK in cells harboring only the BRAF V600E mutation. Therefore, compensatory activation of ERK signalling, which eliminates the inhibitory effects of VEM on ERK, is a key mechanism underlying acquired resistance to VEM in melanoma [12, 13, 14]. Hyper activation of PI3K/AKT is induced by the activation of positive regulators, such as epidermal growth factor receptor (EGFR) and platelet‐derived growth factor *β* (PDGFR), and the loss of negative regulators, such as PTEN [15, 16, 17, 18, 19, 20, 21]. Recently, aberrant upregulation and activation of EphA2, a member of the Eph family of receptor tyrosine kinases (RTKs), has contributed to resistance to VEM in melanoma via the activation of AKT and ERK [22, 23]; this finding suggests that targeting EphA2 is a promising approach for improving VEM efficacy in melanoma.

The upregulation and auto activation of EphA2 activate downstream oncogenic regulators, including PI3K/AKT, MAPK, STAT3, and YAP, which subsequently promote proliferation, metastasis, stemness, and therapeutic resistance in cancers [23, 24, 25, 26, 27]; these findings suggest that targeting EphA2 represents a compelling and promising approach for cancer treatment. Indeed, chemical agents targeting EphA2 have shown satisfactory tumour inhibition efficacy in preclinical and clinical trials [28, 29]. As a potent oncoprotein, EphA2 drives tumourigenicity, metastatic growth, and therapeutic resistance, particularly VEM resistance, in melanoma [30, 31]. For example, EphA2 is upregulated in patients and cell lines with acquired resistance to VEM. Inactivation of EphA2, either through depletion of its expression or inhibition of its activity by specific inhibitors, can significantly increase the sensitivity of VEM‐resistant cells to VEM [32, 33]; this finding suggests that targeted inhibition of EphA2 is a feasible strategy for overcoming VEM resistance in melanoma treatment.

Our group recently demonstrated that the A11, a peptide formed by the fusion of the ANXA1 20–30 amino acid fragment and cell‐penetrating membrane peptide (CPP) exerts potent antitumour effects against nasopharyngeal, gastric, and colorectal carcinomas by promoting EphA2 degradation [34, 35]. Given EphA2's pivotal role in VEM resistance, this study comprehensively evaluated A11's effects on melanoma cell proliferation and VEM sensitivity both in vitro and in vivo. Moreover, we explored whether A11 could promote EphA2 degradation and inhibit the activities of EphA2, AKT, and ERK in melanoma cells. These results demonstrated that A11 could significantly suppress cell proliferation (*p* < 0.05) and prevent VEM resistance in melanoma both in vitro and in vivo. In addition, A11 notably downregulated EphA2 and inhibited the activity of the pS897‐EphA2/AKT/ERK oncogenic signalling pathway in melanoma cells.

## 2 Materials and Methods

### 2.1 TCGA And cBioPortal Databases

The Human Protein Atlas dataset (www.proteinatlas.org) and the cBioPortal for Cancer Genomics (http://cbioportal.org) are used for exploration, visualization, and analysis of comprehensive cancer genomics datasets. These databases are publicly accessible platforms that currently provide data from over 5000 tumour samples across 20 different cancer studies [36]. We also incorporated datasets from The Cancer Genome Atlas (TCGA) [37]. We downloaded RNA‐seq data for 471 melanoma samples in the TCGA database. TCGA and BRAF (V600E) mutation data were combined with wild‐type RNA‐seq data from the Cbioportal database to obtain RNA‐seq data for 309 wild‐type and 162 BRAF (V600E) mutation samples, along with the corresponding clinical data. Data analysis was performed via R 4.0.2.

### 2.2 Cell Lines

The human melanoma cell lines A375 and SK28 and their corresponding VEM‐resistant melanoma cell lines A375‐PR and SK28‐PR were gifted by Professor Chen Xiang's research group of Xiangya Hospital, Central South University. The A375 and SK28 cell lines were grown in DMEM supplemented with 10% FBS (fetal bovine serum). A375‐PR and SK28‐PR cells were grown in DMEM supplemented with 10% FBS and 2 μM VEM. All the cells were cultured in a constant temperature and pressure incubator at 37°C in 5% carbon dioxide. All the cell lines were tested for mycoplasma; however, no mycoplasma was detected.

### 2.3 Western Blotting

Western blotting analyses were conducted following established protocols [34, 38, 39]. In summary, proteins were isolated from the cells with RIPA lysis buffer. Equivalent amounts of protein from each sample were separated by SDS–PAGE and transferred to PVDF membranes. The membranes were then blocked and probed overnight at 4°C with the primary antibody, followed by a 2‐h incubation at room temperature with the HRP‐conjugated secondary antibody. The signals were detected with an enhanced chemiluminescence kit from Roche. Western blotting was performed using three independent biological replicates. GAPDH served as the loading control. Antibodies were sourced as follows: Phospho‐EphA2 (Ser897; #6347), AKT (#2938T), Phospho‐Akt (S473; #4060T), Erk1/2 (#4695), Phospho‐Erk1/2 (P44/42; #4370T), Tanti‐rabbit IgG (#7074), and anti‐mouse IgG (#7076) from Cell Signalling Technology (CST); EphA2 (#sc‐924) from Santa Cruz Biotechnology; and GAPDH (#AC002) from ABclonal.

### 2.4 Immunohistochemistry and Staining Analysis

As previously described, immunohistochemistry (IHC) was conducted on tissue sections that had been fixed with formalin and embedded in paraffin [34, 38, 39]. Briefly, tissue sections were first incubated with a primary antibody at 4°C overnight, followed by incubation with biotinylated secondary antibodies. Afterwards, the sections were treated with the avidin‐biotin peroxidase complex from DAKO for 30 min at room temperature. Finally, the tissue sections were developed with 3,3′‐diaminobenzidine from Sigma–Aldrich as the chromogen and subsequently counterstained with haematoxylin. For the negative control, normal mouse or rabbit immunoglobulin G (IgG) was used instead of the primary antibody. Slides with established positive immunostaining served as positive control samples. Immunohistochemical staining was evaluated and graded by two pathologists who were masked to the clinical and pathological data associated with the samples. Differences were resolved by consensus. The staining intensity was categorized as follows: no staining (score 0), weak staining (score 1), moderate staining (score 2), and strong staining (score 3). The proportion of stained cells, evaluated in a minimum of 500 cells, was classified as no staining = 0, fewer than 30% stained cells = 1, 30%–60% stained cells = 2, and greater than 60% stained cells = 3. The overall staining score for each tissue section was determined by adding the intensity score and the area score, resulting in a score ranging from 0 to 6. A composite score of 3 or below is indicative of low expression levels, whereas a score above 3 suggests high expression levels.

### 2.5 MTT Assay

IC_50_ values of VEM in the melanoma cell and cell proliferation were tested by MTT assay as previously described by us [34, 35], and the experiments were performed in triplicate. Based on the calculated IC_50_ value, 1 μM VEM was selected for subsequent experiments. The concentration of 10 μM for A11 was determined based on dose–response data from our prior studies [30, 31].

### 2.6 Hoechst 33258 Staining for Apoptosis Assay

Following the kit protocol and laboratory experience, cells were grown on coverslips until 50%–80% confluency. After being treated with VEM or/and A11 for 24 h, the cells were fixed with fixative solution for 10 min at room temperature, followed by two washes with PBS. Subsequently, the cells were stained with Hoechst 33,258 staining solution for 5 min in the dark. After two additional PBS washes, the coverslips were mounted onto slides using an anti‐fade mounting medium and immediately examined under a fluorescence microscope for image acquisition.

### 2.7 Plate Colony Formation Assay

The plate colony formation assay, as previously described [34, 35], was utilized to assess cell proliferation and was performed in triplicate.

### 2.8 Tumour Formation Assay in Nude Mice

Four‐week‐old female BALB/c nude mice were purchased from the Experimental Animal Center of Central South University (Changsha, China) and housed under specific pathogen‐free (SPF) conditions. For tumour formation assays, 5 × 10^6^ cells suspended in 200 μL of serum‐free medium were subcutaneously injected into the flanks of mice, with three mice per group. 6 days after tumour inoculation, treatments including A11 and/or VEM were administered via intraperitoneal injection. The A11 peptide was administered at a dose of 10 mg/kg, and VEM was administered at 10 mg/kg, once daily. Tumour sizes were measured daily, and tumour volume was calculated using the formula: Volume = (length × width^2^)/2. After 21 days, mice were euthanized by cervical dislocation. Tumours were harvested, weighed, fixed in 4% paraformaldehyde, and embedded in paraffin for subsequent analysis. Since A11 is composed of the ANXA1 (20–30AA) sequence (EYVQTVKSSKG) fused with the cell‐penetrating peptide CPP (YGRKRRQRRR), CPP was used as a control in all the aforementioned experiments.

### 2.9 Statistical Analysis

The data were statistically analysed with version 22 of the IBM SPSS software package. All experiments were performed in biological triplicate to ensure methodological robustness. The data are expressed as the means ± SDs. For comparisons between two groups, Student's *t*‐test was used. For analysis with multiple comparisons, one‐way ANOVA test was used. All the statistical tests were two‐tailed, and a *p* < 0.05 was taken as the threshold for statistical significance.

## 3 Results

### 3.1 EphA2 Expression Level Is Positively Correlated With BRAF Mutation

**FIGURE 1:**
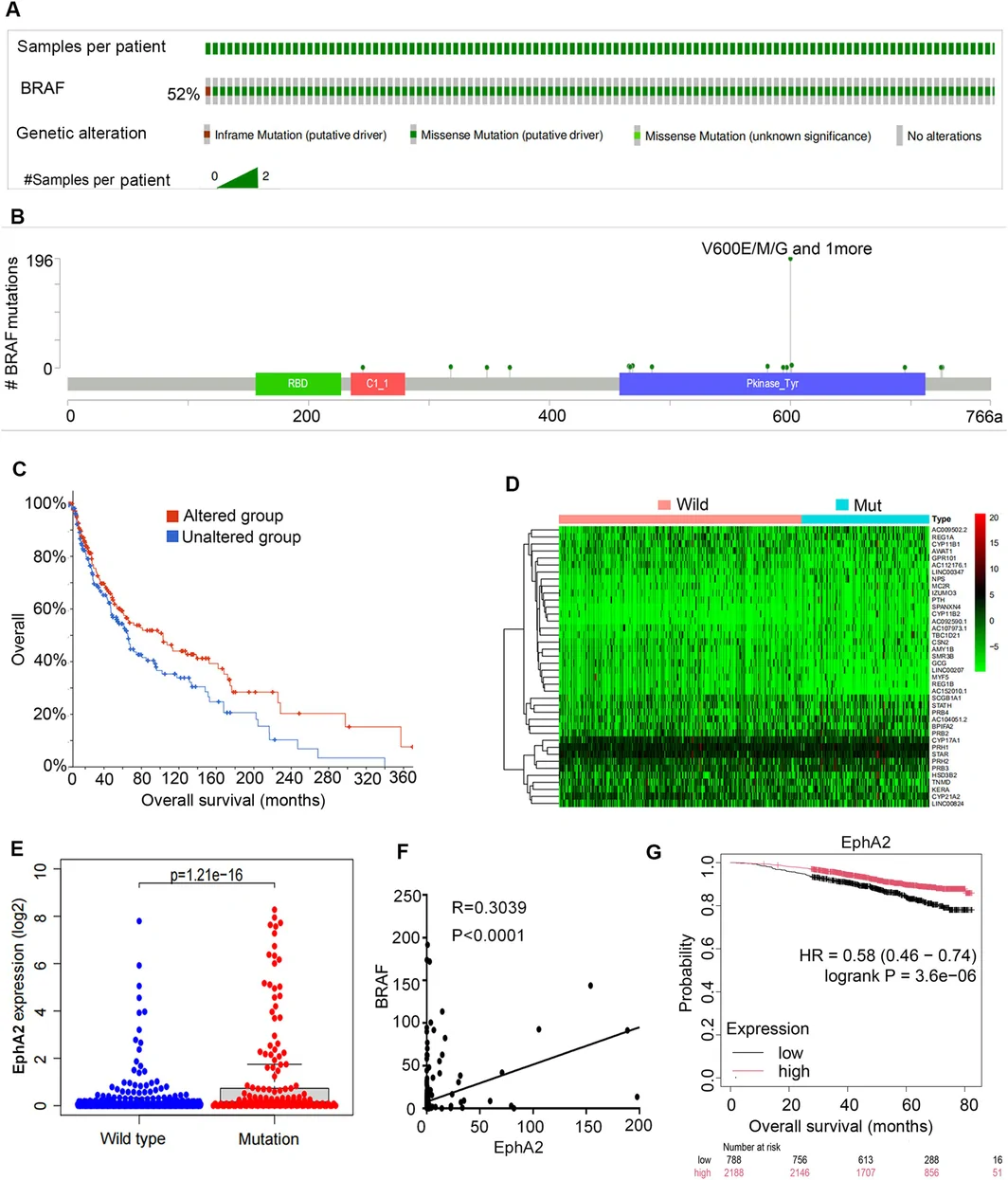
EphA2 expression level was positively correlated with BRAF mutation. (A), The BRAF gene has a mutation rate of 52% in melanoma. (B), The most common BRAF mutation site in melanoma is the V600E/M/G mutation. (C), BRAF mutations are inversely associated with melanoma survival (*p* = 0.0315). (D), Heatmap of differentially expressed genes between the wild‐type BRAF group and BRAF mutant group. (E), EphA2 was differentially expressed between the wild‐type BRAF group and the BRAF mutant group (*p* < 0.05). (F), EphA2 expression was positively correlated with BRAF (V600E) expression (*R* = 0.3039, *p* < 0.0001). (G), TCGA‐SKCM database to investigate the relationship between EphA2 expression levels and overall survival (*p* < 0.05). *p* < 0.05 was considered statistically significant.

Initially, we discovered that the rate of BRAF mutation in SKCM was 52% according to the cBioPortal database (Figure 1A), and the most frequent BRAF mutation site in melanoma was V600E/M/G (Figure 1B). BRAF gene mutation was associated with melanoma patient survival (Figure 1C). We subsequently used R to analyze the differential gene expression patterns between the BRAF mutant group and the wild‐type BRAF group. Heatmaps of differentially expressed genes were generated (Figure 1D). Further analysis revealed that EphA2 was differentially expressed between the BRAF mutant group and the wild‐type BRAF group (Figure 1E) and that EphA2 expression was positively correlated with BRAF mutation (Figure 1F). Therefore, these results demonstrated that the expression level of EphA2 is positively correlated with BRAF mutation.

### 3.2 VEM‐Resistant Melanoma Cells Exhibit Stronger Proliferation Than Parental Cells

**FIGURE 2:**
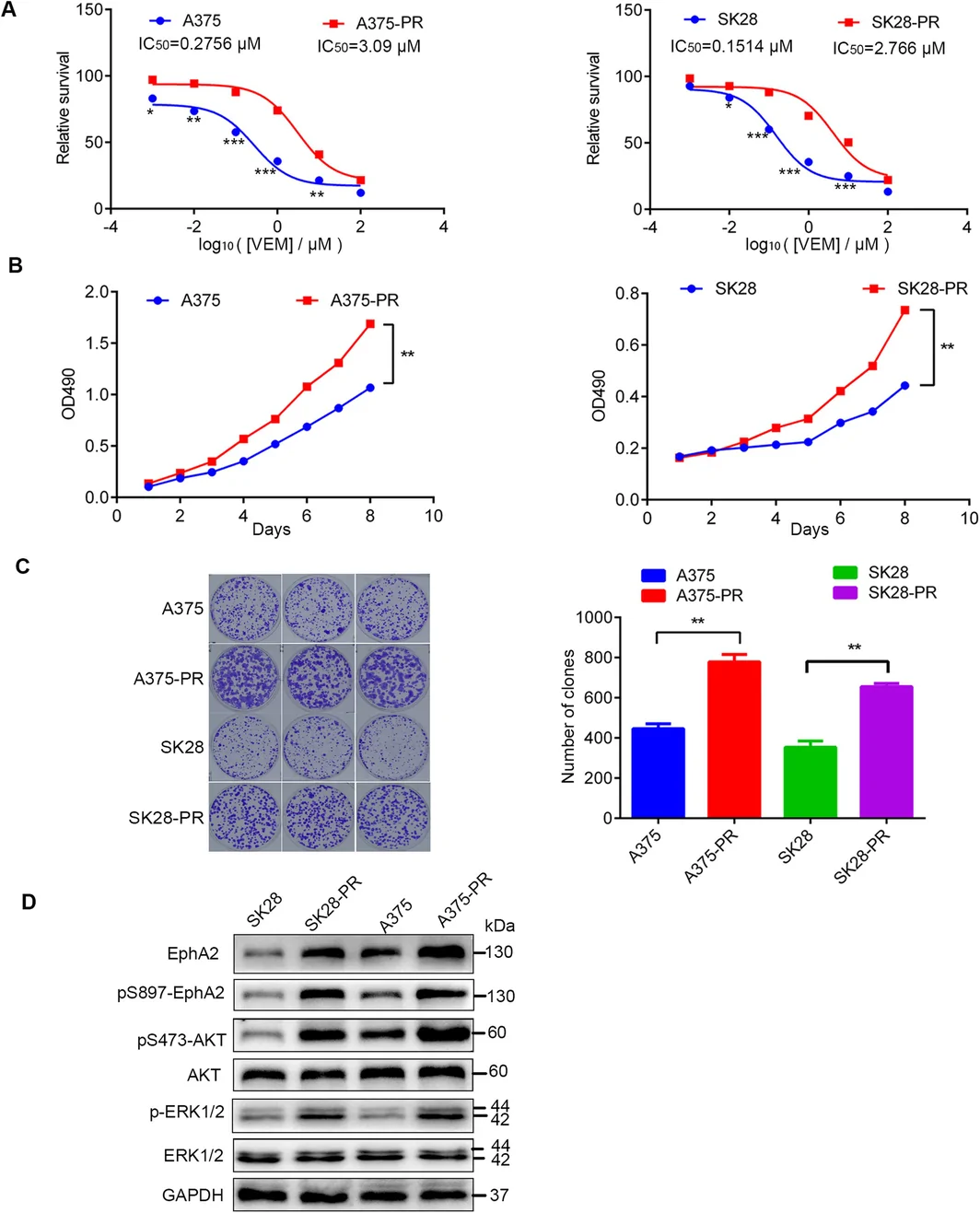
VEM‐resistant melanoma cells proliferate more than parental cells. (A), MTT assay showing the IC_50_ values of VEM in the VEM‐sensitive melanoma cell lines A375 and SK28 and their corresponding VEM‐resistant melanoma cell lines A375‐PR and SK28‐PR. B‐C, MTT assay (B) and plate colony formation assay (C) showing the proliferation of the VEM‐sensitive melanoma cell lines A375 and SK28 and their corresponding VEM‐resistant melanoma cell lines A375‐PR and SK28‐PR. (D), Western blotting showing the expression of EphA2, p‐EphA2 (S897), p‐AKT (S473), and p‐ERK1/2 (T202/Y204) in the VEM‐sensitive melanoma cell lines A375 and SK28 and their corresponding VEM‐resistant melanoma cell lines A375‐PR and SK28‐PR. GAPDH was used as a loading control. The numbers represent the means ± SDs (*n* = 3 biological replicates/group). Statistical significance was determined by two‐tailed unpaired Student's *t*‐test (Figure 2A–C). Exact *p*‐values are annotated on graphs: *p < 0.05; **p < 0.01; ***p < 0.001; ns: No statistical significance.

To reveal the mechanisms underlying VEM resistance in melanoma, we first compared the differences in proliferation between VEM‐resistant and parental melanoma cells with MTT and plate clone survival assays. As shown in Figure 2A, VEM‐resistant cells had greater IC_50_ values than parental cells. Moreover, the proliferation of VEM‐resistant melanoma cells was notably greater than that of parental cells, as shown by the results of the MTT and plate colony survival assays (Figure 2B, C). In addition, compared with those in parental cells, the level of EphA2 and the activation of p‐EphA2 (S897), p‐AKT (S473), and p‐ERK1/2 (T202/Y204) were increased in VEM‐resistant melanoma cells (Figure 2D), with ANXA1 expression levels consistent with those of EphA2 (Figure S1). These results confirmed that VEM‐resistant melanoma cells exhibited stronger proliferation due to the increased expression of EphA2 and pS897‐EphA2/AKT/ERK oncogenic signalling pathway activation.

### 3.3 A11 Suppresses the Proliferation of Both VEM‐Resistant and Parental Melanoma Cells

**FIGURE 3:**
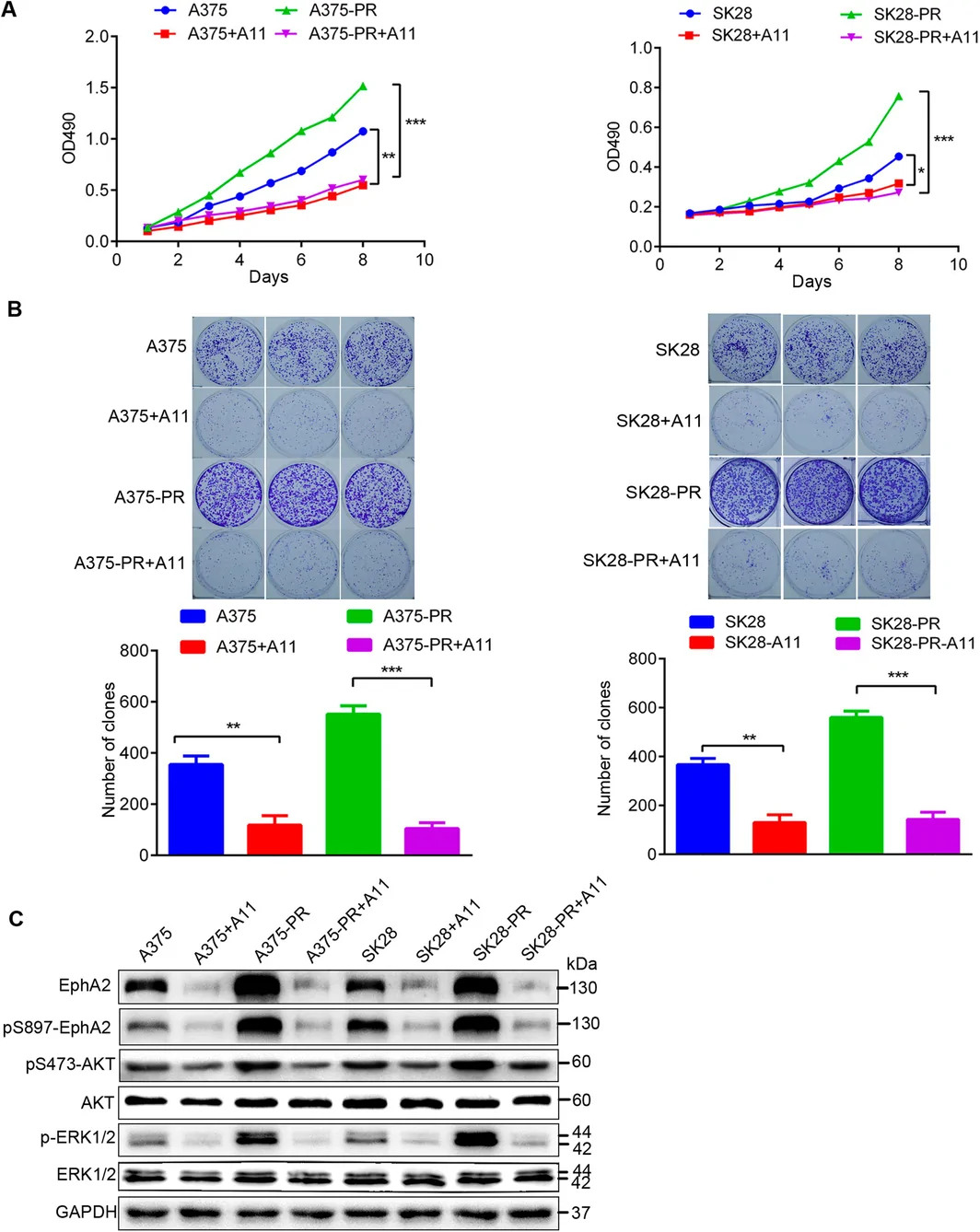
A11 suppresses the proliferation of both VEM‐resistant and parental melanoma cells. (A‐B), MTT assay (A) and plate colony formation assay (B) showing that A11 significantly inhibited the proliferation of VEM‐resistant A375‐PR and SK28‐PR melanoma cells and VEM‐sensitive A375 and SK28 melanoma cells. (C), Western blotting showing that A11 treatment significantly decreased the levels of total EphA2, p‐EphA2 (S897), p‐AKT (S473), and p‐ERK1/2 (T202/Y204) in both VEM‐resistant and parental melanoma cells. GAPDH was used as a loading control. The numbers represent the means ± SDs (*n* = 3 biological replicates/group). Statistical significance was determined by two‐tailed unpaired Student's *t*‐test (Figure 3A,B). Exact *p*‐values are annotated on graphs: *p < 0.05; **p < 0.01; ***p < 0.001; ns: No statistical significance.

Preliminary research had confirmed that A11 exerts antitumour effects by promoting the degradation of EphA2 [34, 35]. Additionally, our previous studies revealed that the proliferation of melanoma cells was positively correlated with the expression level of EphA2. Therefore, we scientifically hypothesized that A11 may inhibit the viability and proliferation of melanoma cells. Consistently, A11 (10 μM) markedly inhibited the proliferation of both VEM‐resistant and parental melanoma cells, as demonstrated by the results of the MTT and plate colony survival assays (Figure 3A,B). Moreover, A11 treatment induced pronounced downregulation of the level of total EphA2 and suppressed the activity of p‐EphA2 (S897), p‐AKT (S473), and p‐ERK1/2 (T202/Y204) in both VEM‐resistant and parental melanoma cells (Figure 3C). These results confirmed that A11 could suppress the proliferation of melanoma cells by downregulating EphA2 and suppressing the activity of the pS897‐EphA2/AKT/ERK oncogenic signalling pathway.

### 3.4 A11 Enhances the Sensitivity of Melanoma Cells to VEM In Vitro

**FIGURE 4:**
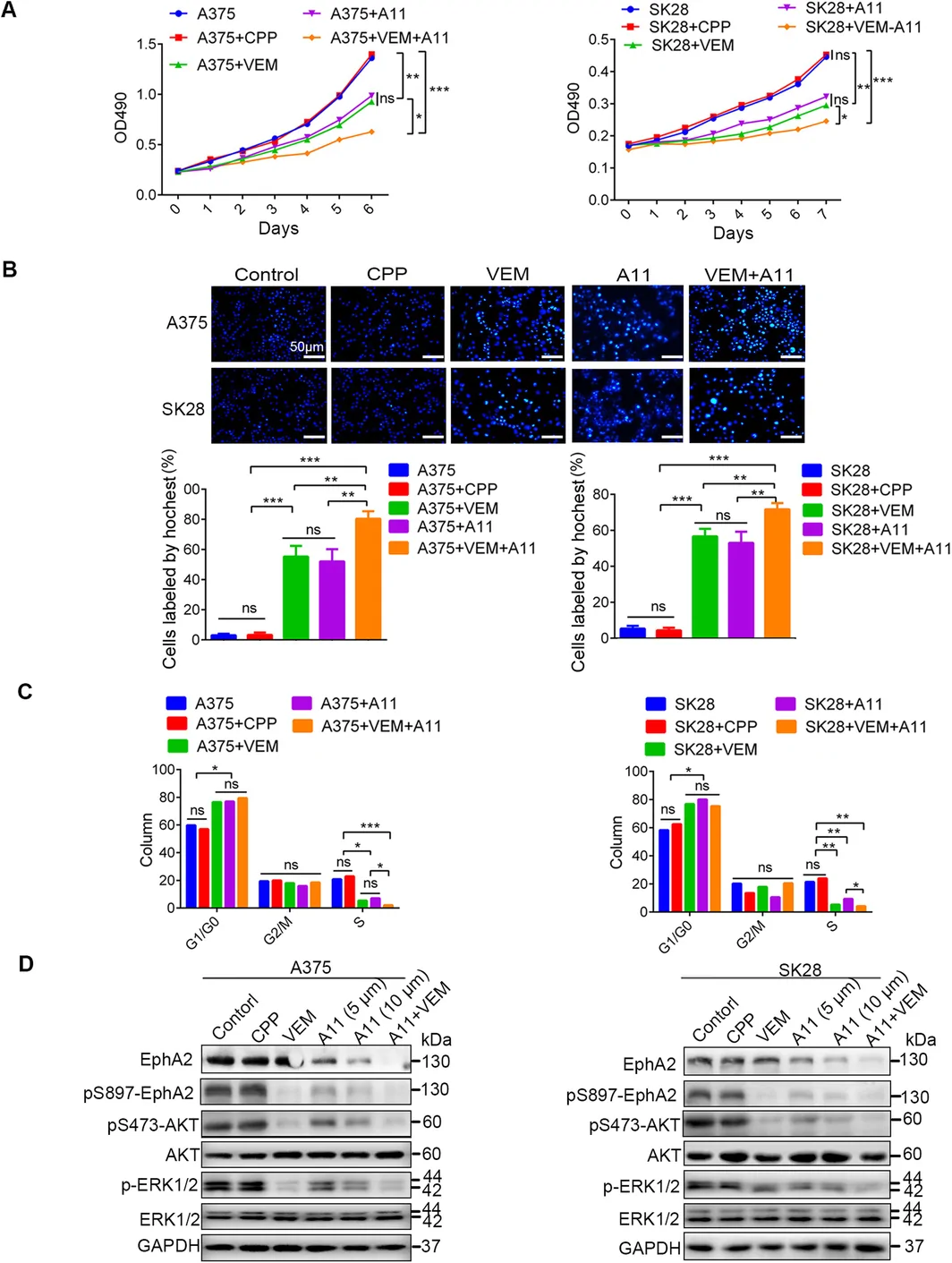
The effects of A11 combined with VEM on the proliferation, apoptosis, and cell cycle of VEM‐sensitive melanoma cells in vitro. (A), MTT assay showing the effects of A11, VEM, or A11 combined with VEM on the proliferation of A375 and SK28 melanoma cells. (B), Hoechst 33,258 staining showing the effects of A11, VEM, or A11 combined with VEM on the apoptosis of A375 and SK28 melanoma cells. (C), Flow cytometry showing the effects of A11, VEM, or A11 combined with VEM on the cell cycle distribution of melanoma cells. (D), Western blotting showing the effects of A11, VEM, or A11 combined with VEM on the level of total EphA2, p‐EphA2 (S897), p‐AKT (S473), and p‐ERK1/2 (T202/Y204) in A375 and SK28 melanoma cells. GAPDH was used as a loading control. The numbers represent the means ± SDs (*n* = 3 biological replicates/group). Statistical significance was determined by one‐way ANOVA (Figure 4A–C). Exact *p*‐values are annotated on graphs: *p < 0.05; **p < 0.01; ***p < 0.001; ns: No statistical significance.

**FIGURE 5:**
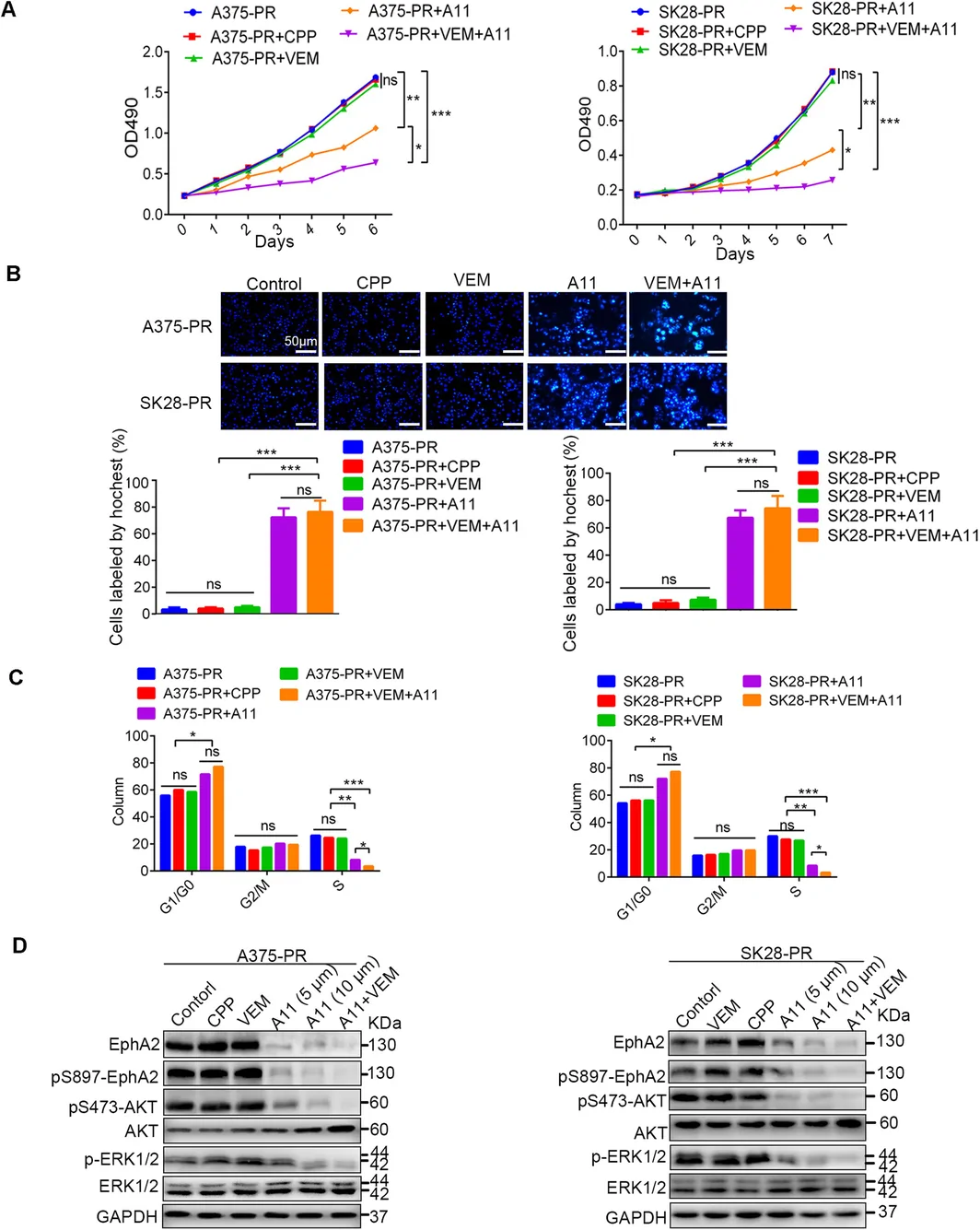
The effects of A11 combined with VEM on the proliferation, apoptosis, and cell cycle progression of VEM‐resistant melanoma cells in vitro. (A), MTT assay showing the effects of A11, VEM, or A11 combined with VEM on the proliferation of the VEM‐resistant melanoma cell lines A375‐PR and SK28‐PR. (B), Hoechst 33,258 staining showing the effects of A11, VEM, or A11 combined with VEM on the apoptosis of VEM‐resistant A375‐PR and SK28‐PR melanoma cells. (C), Flow cytometry showing the effects of A11, VEM, or A11 combined with VEM on the cell cycle progression of VEM‐resistant A375‐PR and SK28‐PR melanoma cells. (D), Western blotting showing the effects of A11, VEM, or A11 combined with VEM on the level of total EphA2, p‐EphA2 (S897), p‐AKT (S473), and p‐ERK1/2 (T202/Y204) in VEM‐resistant A375‐PR and SK28‐PR melanoma cells. GAPDH was used as a loading control. The numbers represent the means ± SDs (*n* = 3 biological replicates/group). Statistical significance was determined by one‐way ANOVA (Figure 5A–C). Exact *p*‐values are annotated on graphs: *p < 0.05; **p < 0.01; ***p < 0.001; ns: No statistical significance.

VEM is an effective therapy for BRAF (V600E)‐mutant melanoma but is not effective in treating VEM‐resistant melanoma cells. Therefore, we analysed the effects of A11 combined with VEM treatment on the proliferation of melanoma cells by MTT, Hoechst 33,258 staining, and cell cycle analyses. These experiments were performed after the cells were treated with A11 (10 μM) and/or VEM (1 μM). A11 and VEM significantly (*p* < 0.05 for all comparisons) and equally suppressed the viability and induced the apoptosis and cell cycle arrest of parental melanoma cells (Figure 4A–C). Moreover, A11 and VEM exerted notable synergetic inhibitory effects on parental melanoma cells (Figure 4A–C). More importantly, our research revealed that A11 showed significant inhibitory effects, while VEM exerted no inhibitory effects on VEM‐resistant melanoma cells (Figure 5A–C). The combined application of A11 and VEM synergistically inhibited proliferation and caused cell cycle arrest (Figure 5A,C). A11 monotherapy significantly promotes apoptosis in VEM‐resistant melanoma cells, while the combination of A11 and vemurafenib does not lead to a further significant increase in apoptosis (Figure 5B). Based on existing literatures [40, 41, 42, 43, 44, 45, 46, 47, 48], we reasonably infer that this phenomenon may be attributed to the fact that A11 has already triggered ‘apoptosis saturation’, and that other non‐apoptotic forms of cell death may also be involved. Moreover, the IC₅₀ values of the A11 and VEM combination therapy were significantly lower than those of A11 monotherapy across all tested cell lines (Figure S2). Thus, these results suggested that A11 could sensitize melanoma cells to VEM in vitro.

Furthermore, the level of total EphA2 and the activity of the pS897‐EphA2/AKT/ERK oncogenic signalling pathway were also analysed by Western blotting. The results showed that VEM treatment alone notably inhibited p‐EphA2 (S897), p‐AKT (S473), and p‐ERK1/2 (T202/Y204) expression but did not inhibit total EphA2 expression in parental cells (Figure 4D). Consistent with the known mechanisms [12, 13, 14], the inhibitory effects of VEM on the pS897‐EphA2/AKT/ERK oncogenic signalling pathway were abolished in VEM‐resistant melanoma cells (Figure 5D). When VEM was combined with A11, the inhibitory effects of VEM on the pS897‐EphA2/AKT/ERK oncogenic signalling pathway were reversed, possibly due to the downregulation of total EphA2 in VEM‐resistant melanoma cells (Figure 5D).

### 3.5 A11 Increases the Sensitivity of Melanoma Cells to VEM In Vivo

**FIGURE 6:**
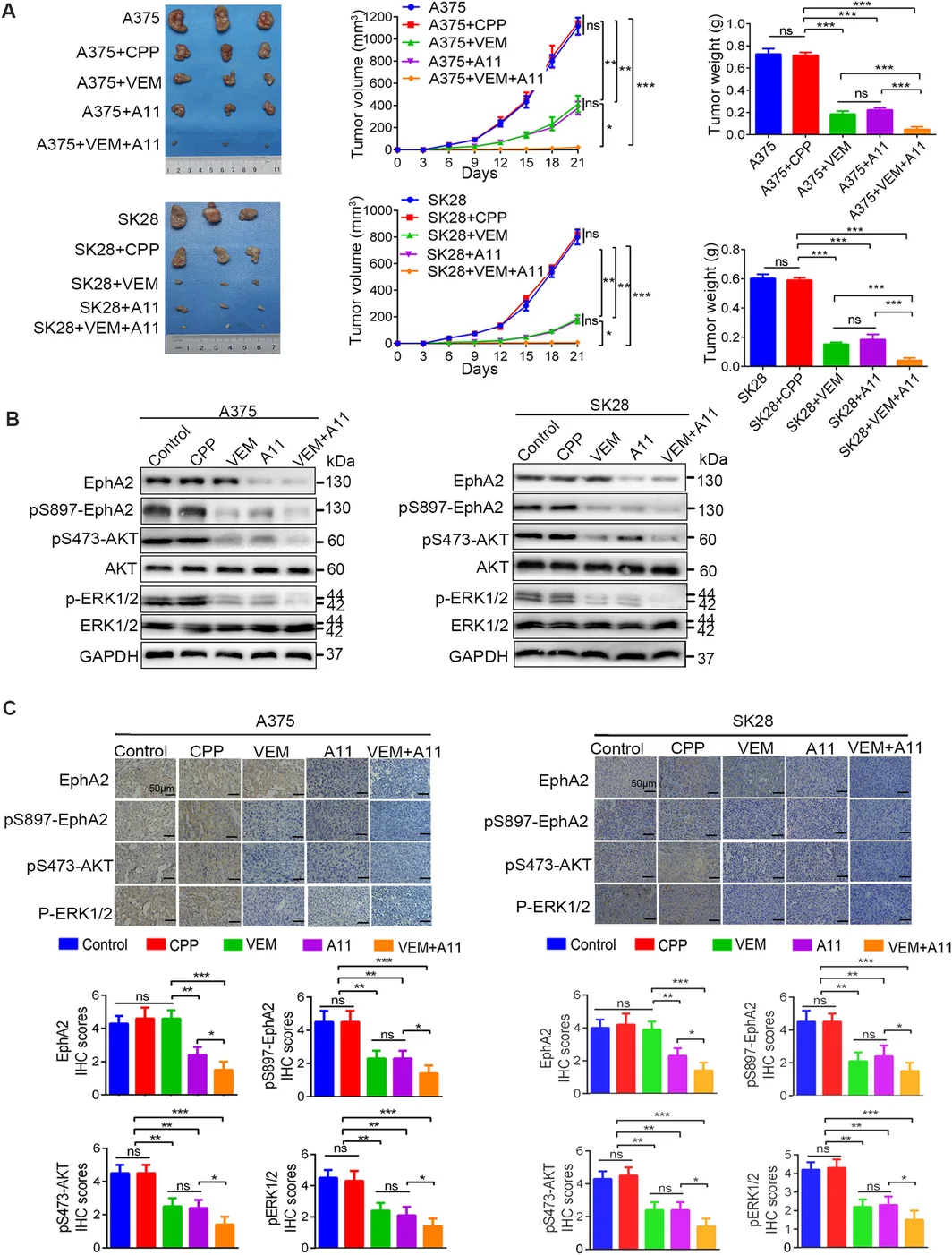
The effect of A11 combined with VEM on the growth of VEM‐sensitive melanoma cells in vivo. (A), Subcutaneous xenografts derived from A375 and SK28 cells were harvested from each mouse and photographed prior to treatment, and curves and histograms show the volume and weight of the subcutaneous xenografts in each group (three mice), respectively. (B), Western blotting showing the level of total EphA2, p‐EphA2 (S897), p‐AKT (S473), and p‐ERK1/2 (T202/Y204) in the subcutaneous xenografts. GAPDH was used as a loading control. (C), IHC showing the level of total EphA2, p‐EphA2 (S897), p‐AKT (S473), and p‐ERK1/2 (T202/Y204) in the subcutaneous xenografts. A representative IHC image is displayed above, and the quantitative data are presented below. The numbers represent the means ± SDs (*n* = 3 biological replicates/group). Statistical significance was determined by one‐way ANOVA (Figure 6A,C). Exact *p*‐values are annotated on graphs: *p < 0.05; **p < 0.01; ***p < 0.001; ns: No statistical significance.

**FIGURE 7:**
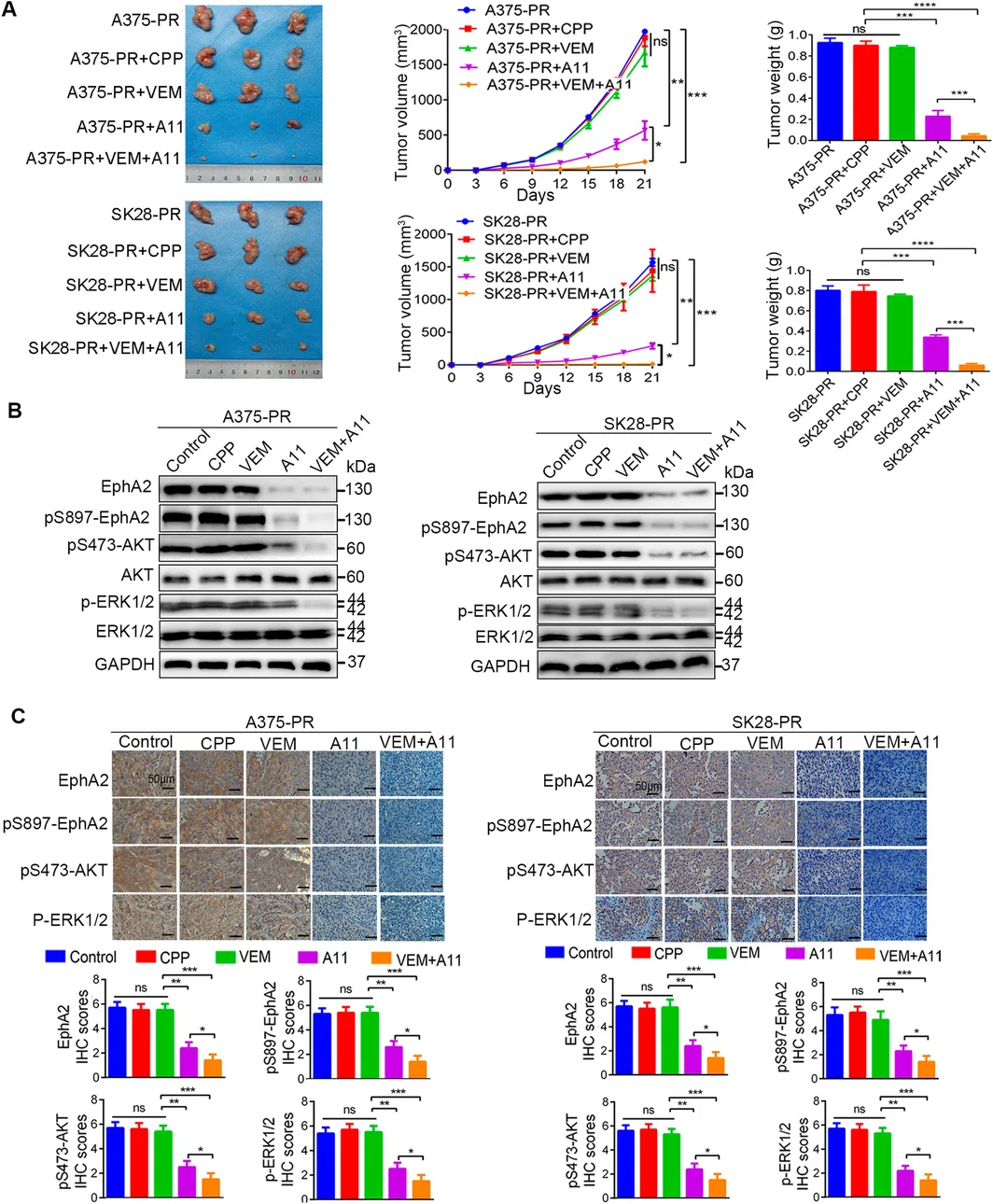
The effect of A11 combined with VEM on the growth of VEM‐resistant melanoma cells in vivo. (A), Subcutaneous xenografts of A375‐PR and SK28‐PR cells were harvested from each mouse and photographed prior to treatment, and curves and histograms show the volume and weight of the subcutaneous xenografts in each group (three mice), respectively. (B), Western blotting showing the level of total EphA2, p‐EphA2 (S897), p‐AKT (S473), and p‐ERK1/2 (T202/Y204) in the subcutaneous xenografts. GAPDH was used as a loading control. (C), IHC showing the level of total EphA2, p‐EphA2 (S897), p‐AKT (S473), and p‐ERK1/2 (T202/Y204) in the subcutaneous xenografts. A representative IHC image is displayed above, and the quantitative data are presented below. The numbers represent the means ± SDs (*n* = 3 biological replicates/group). Statistical significance was determined by one‐way ANOVA (Figure 7A,C). Exact *p*‐values are annotated on graphs: *p < 0.05; **p < 0.01; ***p < 0.001; ns: No statistical significance.

The synergetic effects of A11 and VEM were subsequently validated in vivo in a subcutaneous tumour model. Consistently, A11 (10 μM) and VEM (1 μM) significantly (*p* < 0.05 for all comparisons) and fairly suppressed the in vivo proliferation of parental melanoma cells, as demonstrated by the volume and weight of the xenograft tumours (Figure 6A). Similarly, A11 alone exerted significant inhibitory effects, while VEM alone exerted no inhibitory effects on the in vivo proliferation of VEM‐resistant melanoma cells (Figure 7A). However, the combined injection of A11 and VEM synergistically suppressed the in vivo proliferation of VEM‐resistant melanoma cells (Figure 7A). These results suggested that A11 could sensitize melanoma cells to VEM in vivo.

Western blotting and IHC staining were used to assess total EphA2 levels and pS897‐EphA2/AKT/ERK pathway activity in xenograft tumours. Consistent with the in vitro results, A11 treatment induced a pronounced reduction in the levels of total EphA2, p‐EphA2 (S897), p‐AKT (S473), and p‐ERK1/2 (T202/Y204) in both resistant (Figure 7B,C) and parental (Figure 6B,C) xenografts. VEM monotherapy did not significantly reduce these targets in resistant xenografts, whereas the A11 + VEM combination markedly and synergistically suppressed their expression (Figure 7B,C). These results suggested that A11 promoted EphA2 degradation and suppressed the pS897‐EphA2/AKT/ERK oncogenic pathway in resistant melanoma.

### 3.6 A11 Peptide Enhances VEM Sensitivity in Melanoma Cells Through Targeted EphA2 Suppression

To validate that A11 specifically targets EphA2 to sensitize melanoma cells to VEM and reverse resistance, we performed rescue experiments in parental (A375, SK28) and VEM‑resistant (A375‑PR, SK28‑PR) cell lines. MTT assays revealed that EphA2 overexpression significantly counteracted A11‐induced proliferation suppression in both VEM‐sensitive (A375, SK28) and VEM‐resistant (A375‐PR, SK28‐PR) cell lines (Figure S3A; Figure S4A). Hoechst 33,258 staining confirmed reversal of A11‐triggered apoptosis upon EphA2 overexpression (Figure S3B; Figure S4B). Western blot analysis showed that EphA2 overexpression significantly counteracted A11‐induced total EphA2, p‐EphA2 (S897), p‐AKT (S473), and p‐ERK1/2 (T202/Y204) levels (Figure S3C; Figure S4C). Moreover, RNA‐seq experimental analyses reveal that under A11 treatment, the EphA2/AKT/ERK signalling pathway is significantly suppressed, whereas no inhibition of other known resistance pathways, such as PDGFRβ, EGFR, or YAP, was observed (Figure S5). These results definitively establish that A11 enhances the sensitivity of melanoma cells to VEM by inhibiting EphA2 and its downstream signalling pathway.

## 4 Discussion

Vemurafenib resistance remains a primary challenge for achieving sustained efficacy in melanoma therapy. This study demonstrates for the first time that the ANXA1‐derived peptide A11 significantly reverses vemurafenib resistance in melanoma both in vitro and in vivo. The underlying mechanism involves targeted degradation of EphA2 and inhibition of its downstream oncogenic pS897‐EphA2/AKT/ERK signalling pathway. While our previous research has focused on the oncogenic mechanisms of EphA2 and the anticancer effects of the A11 peptide in nasopharyngeal carcinoma and gastrointestinal tumours, this investigation specifically addresses the clinically urgent yet underexplored area of acquired vemurafenib resistance in melanoma.

Supported by public database analysis, we first confirmed elevated EphA2 expression in BRAF‐mutant versus wild‐type melanoma (*p* < 0.05) and its correlation with poor overall survival (*p* < 0.05). Biological comparison of VEM‐sensitive (A375, SK28) and resistant (A375‐PR, SK28‐PR) cells revealed that resistant lines exhibited substantially higher VEM IC_50_ values, accelerated proliferation rates, and increased protein expression of EphA2, p‐EphA2 (S897), p‐Akt (S473), and p‐ERK1/2(T202/Y204). Critically, A11 monotherapy or combined with VEM significantly inhibited proliferation and induced apoptosis in resistant cells, with the combination demonstrating superior efficacy versus A11 alone (*p* < 0.05). Mechanistically, both treatment modalities substantially reduced expression of EphA2, p‐EphA2 (S897), p‐Akt (S473), and p‐ERK1/2 (T202/Y204), confirming pathway suppression as the underlying mechanism.

In the melanoma xenograft model, VEM monotherapy exhibited negligible efficacy against VEM‐resistant tumours, whereas A11 alone significantly suppressed tumour growth. The A11 and VEM combination demonstrated superior antitumour activity compared to A11 monotherapy (*p* < 0.05). Importantly, prior toxicology assessments [34, 35] confirmed no treatment‐related toxicity for A11, including stable body weight (< 5% fluctuation), absence of behavioural abnormalities, and preserved histoarchitecture of vital organs (heart, liver, lungs, kidneys) without inflammatory infiltrates or necrosis, attributable to A11's selective targeting of tumour‐enriched EphA2.

Based on our experimental findings, VEM monotherapy shows no effect on EphA2 signalling in resistant cells, whereas its combination with A11 results in synergistic suppression of proliferation. This suggests that the cooperative anti‐proliferative effect extends beyond EphA2 inhibition. We propose that A11‐mediated degradation of EphA2 disrupts this pivotal hub of the resistance network. EphA2 serves as a signalling nexus that, when overexpressed, activates multiple downstream survival pathways, including PI3K/AKT, STAT3 [23, 24, 26]. By eliminating EphA2, A11 not only attenuates these parallel survival signals but may also reverse the resistant cell state, thereby restoring sensitivity to MAPK pathway inhibition. Thus, in A11‐primed cells, VEM can more effectively suppress residual MAPK activity, establishing a multi‐pronged blockade of survival signalling. This synergistic model offers new perspectives for overcoming targeted therapy resistance, with the elucidation of its detailed mechanisms representing a key direction for our future research.

Our findings position A11 as a promising therapeutic strategy, but its advantages are best understood in the context of existing EphA2‐targeting modalities. Current approaches, such as small‐molecule inhibitors (e.g., ALW‐II‐41‐27) and antibody‐based therapies, often face significant challenges. These include off‐target effects leading to high cytotoxicity, limited penetration into solid tumours, and the eventual development of drug resistance [26, 49, 50]. In contrast, the A11 peptide employs a distinct mechanism. A11 is designed to mimic the native ligand interface and functions by specifically disrupting the ANXA1‐EphA2 interaction. This unique action promotes Cbl‐mediated ubiquitination and subsequent degradation of the EphA2 protein, offering a targeted method to deplete oncogenic EphA2 [34]. This mechanism underlies A11's high specificity and reduced off‐target toxicity. Furthermore, the conjugation of a cell‐penetrating peptide (CPP) efficiently addresses the perennial challenge of intracellular delivery for macromolecular drugs, setting it apart from delivery‐limited antibody therapies.

Although there have been no reports on the use of peptides in the treatment of VEM‐resistant melanoma, great progress has been made in the study of the use of peptides in the treatment of tumours. Moreover, a variety of peptides have been used to treat tumours. Compared with traditional chemotherapeutic drugs, polypeptide drugs are characterized by strong targeting ability, good effects, and low toxicity. Compared with other small‐molecule inhibitors, polypeptide drugs are easy to synthesize and modify, and they have good biocompatibility. Compared with protein drugs, polypeptide drugs are characterized by low molecular weights, low immunogenicity and high biological activity; additionally, their synthesis is inexpensive, and they are easy to purify [51, 52]. Therefore, the peptide‐based approach exemplified by A11 represents a highly promising new strategy for overcoming drug resistance in melanoma.

## 5 Conclusion

In this study, our findings indicate that A11 can markedly inhibit the proliferation and reverse the VEM resistance of melanoma cells by downregulating EphA2 and subsequently suppressing the pS897‐EphA2/AKT/ERK oncogenic signalling pathway.

## Author Contributions

**Xiao‐Pu Huang:** data curation, investigation, writing – original draft. **Juan Feng:** writing – review and editing, funding acquisition, formal analysis. **Ming Zhang:** validation. **Wei Huang:** conceptualization, project administration. **Shan‐Shan Lu:** methodology, supervision. **Zhi‐Qiang Xiao:** conceptualization. **Zhi‐Hai Xie:** resources. **Hong Yi:** conceptualization, funding acquisition, project administration, writing – review and editing.

## Funding

This work was supported by the following research grants: National Natural Science Foundation of China (82272798, 82170552, 82203161); Natural Science Foundation of Hunan Province (2021JJ31120, 2023JJ40815, 2026JJ50070); Scientific Research Fund of Hunan Provincial Education Department (21A0008); Scientific Research Launch Project for new employees of the Second Xiangya Hospital of Central South University.

## Ethics Statement

All animal experimental procedures were performed in accordance with the Guide for the Care and Use of Laboratory Animals of Xiangya Hospital of Central South University, with the approval of the Institutional Animal Ethics Committee. All animal studies also complied with the ARRIVE guidelines and the AVMA euthanasia guidelines 2020.

## Consent

The authors have nothing to report.

## Conflicts of Interest

The authors declare no conflicts of interest.

## Supporting information


**Figure S1:** The expression levels of both EphA2 and ANXA1 were significantly upregulated in VEM‐resistant melanoma cells compared with those in parental cells. Western blotting showing the expression level of ANXA1 and EphA2 in both VEM‐sensitive and VEM‐resistant melanoma cells. GAPDH was used as a loading control.

**Figure S2:** The IC_50_ value of the A11 and VEM combination therapy was significantly lower than of A11 monotherapy in both VEM‐resistant and parental melanoma cells. MTT assay showing the IC_50_ values of and values of A11 and its combination with VEM in melanoma cell lines. Statistical significance was determined by two‐tailed unpaired Student's *t*‐test. Exact *p*‐values are annotated on graphs: *p < 0.05; **p < 0.01; ***p < 0.001; ns: no statistical significance.

**Figure S3:** EphA2 overexpression via plasmid transfection reversed A11‐induced phenotypic alterations in VEM‐sensitive melanoma cells. (A), MTT assay showing that EphA2 overexpression reversed A11‐induced proliferation inhibition of the VEM‐sensitive melanoma cell lines A375 and SK28. (B), Hoechst 33,258 staining showing that EphA2 overexpression reversed A11‐induced apoptosis of the VEM‐sensitive melanoma cell lines A375 and SK28. (C), Western blotting showing that EphA2 overexpression reversed A11‐induced phosphorylation levels at key signalling nodes (pS897‐EphA2, pS473‐AKT, p‐ERK1/2). GAPDH was used as a loading control. The numbers represent the means ± SDs. *p < 0.05; **p < 0.01; ***p < 0.001; ns: no statistical significance.

**Figure S4:** EphA2 overexpression via plasmid transfection reversed A11‐induced phenotypic alterations in VEM‐resistant melanoma cells. (A), MTT assay showing that EphA2 overexpression reversed A11‐induced proliferation inhibition of the VEM‐resistant A375‐PR and SK28‐PR melanoma cells. (B), Hoechst 33,258 staining showing that EphA2 overexpression reversed A11‐induced apoptosis of the VEM‐resistant A375‐PR and SK28‐PR melanoma cells. (C), Western blotting showing that EphA2 overexpression reversed A11‐induced phosphorylation levels at key signalling nodes (pS897‐EphA2, pS473‐AKT, p‐ERK1/2). GAPDH was used as a loading control. The numbers represent the means ± SDs. *p < 0.05; **p < 0.01; ***p < 0.001; ns: no statistical significance.

**Figure S5:** Heatmap showing expression of differentially expressed genes in A375‐PR cells treated with or without A11, based on RNA‐seq. Colour scale indicates expression levels (red, upregulated; blue, downregulated).

## Data Availability

The data that support the findings of this study are available from the corresponding author upon reasonable request.
